# How Servant Leadership Sparks Feedback-Seeking Behavior: A Moderated Mediation Model

**DOI:** 10.3389/fpsyg.2021.748751

**Published:** 2021-11-03

**Authors:** Dong Qin, Yan Xu, Chaoping Li, Xue Meng

**Affiliations:** School of Public Administration and Policy, Renmin University of China, Beijing, China

**Keywords:** servant leadership, feedback-seeking behavior, *moqi* with supervisors, traditionality, social information processing theory

## Abstract

Drawing upon social information processing theory, we propose that *moqi* with supervisors mediates the relationship between servant leadership and follower feedback-seeking behavior. Subordinates’ traditionality plays a moderating role in this process. A total of 440 Chinese working adults responded to the two-wave questionnaire survey in paper and pencil forms. Correlation analyses, mediation analysis, and moderated mediation analysis was performed through R and SPSS PROCESS Macro. The results revealed that servant leadership positively correlates with followers’ feedback-seeking behavior via *moqi* with supervisors. Moreover, these indirect effects of servant leadership were moderated by traditionality, such that servant leadership had weaker relations with feedback-seeking behavior when traditionality was higher (vs. lower). Theoretical contributions and practical implications, limitations and suggestions for further study were discussed.

## Introduction

As labor market conditions rapidly change with the development of the economy and society, both organizational environment and requirements for employees are increasingly complex in the workplace. When employees are uncertain about their role or performance, they would actively seek relevant feedback information instead of waiting passively to receive it (Zhang et al., 2017). Information collected from feedback helps seekers to improve in many ways (Anseel et al., 2015; Ashford et al., 2016; Sherf and Morrison, 2020). For example, seeking feedback can help newly hired employees clearly understand their role expectations, assist older and experienced employees improve their performance (Ashford and Tsui, 1991; Gong et al., 2017). Given the valuable information gathered through feedback-seeking contributes to employees’ development and improvement, it is important to investigate its antecedents trying to identify ways to encourage it (e.g., Qian et al., 2017; Sherf and Morrison, 2020).

Researchers have found that contextual factors and individual differences are crucial antecedents of employees’ feedback-seeking behavior (Ashford et al., 2003; Anseel et al., 2015). Among these contextual factors, leaders play the dominant role in followers’ work lives (Chen et al., 2015). However, how managers can encourage employees’ feedback-seeking behavior remains further developed. A few recent studies of feedback-seeking attempted to demonstrate leaders’ impact on employees’ feedback-seeking, such as transformational leadership (Levy et al., 2002; Wang et al., 2016), and ethical leadership (Qian et al., 2017; Moss et al., 2020). Following this research line, we focus on a more moral and ethical style of leadership (Lemoine et al., 2018), named servant leadership. Servant leadership focuses on putting the needs of followers and stakeholders first (Greenleaf, 1970, 1977), and it could better explain a wide range of outcomes compared with ethical, authentic, and transformational leadership (Hoch et al., 2018; Lee et al., 2020). Researchers have investigated its significant influence on employees’ behavior job-related outcomes, such as engagement (De Clercq et al., 2014), organizational citizenship behavior (OCB) (Liden et al., 2014), task performance (Chen et al., 2015), and creativity (Yoshida et al., 2014). A series of literature reviews and meta-analyses also have evidenced servant leadership is positively related to followers’ job-related outcomes (e.g., Eva et al., 2019; Li et al., 2021; Zhang et al., 2021). Given servant leaders prioritize follower needs over self-interest, they would provide credible and valuable feedback to their followers, and strive to help employees to grow and realize their full potential. However, whether and how employees’ feedback-seeking behavior was encouraged by servant leadership remains unclear, it is of both theoretical and empirical importance to investigate the relationship between them.

The present study attempts to address these gaps by examining the impact of servant leadership on employees’ feedback-seeking behavior. Based on social information processing theory (SIPT, Salancik and Pfeffer, 1978) and the feedback-seeking behavior literature, we provide a social information processing model to explain the positive association between servant leadership and feedback-seeking behavior. Social information processing theory is widely used to explain the influence of servant leaders on their subordinates’ work behaviors and the impact of contextual factors on feedback-seeking behavior (e.g., Chuang et al., 2014; Lu et al., 2019). In addition, servant leadership emphasizes one-on-one prioritizing of follower individual needs and interests, and attach importance to doing good to others (Eva et al., 2019). The subordinates may perceive feedback from servant leaders as more credible and valuable, and thus they are more likely and frequently seek feedback from servant leaders (Anseel et al., 2015; Huang et al., 2018).

According to social information processing theory, followers use information from work contexts to understand leaders’ expectations and decide how to behave, *moqi* with supervisors may serve as a mediator through which servant leadership positively influences feedback-seeking behavior. *Moqi* with supervisors is a Chinese construct defined as “subordinates’ implicit understanding of their superiors’ work-related expectations, requirements, and intentions” (Zheng et al., 2019). It helps subordinates form a better understanding of their expectations and performance through the interaction with leaders so that they could have confidence and psychological safety to seek feedback without hesitation (Chughtai, 2016). Therefore, we propose that *moqi* with supervisors serves as a mediator through which servant leadership positively influences feedback-seeking behavior.

As we discussed before, contextual factors and individual differences are crucial antecedents of employees’ feedback-seeking behavior. It is important for researchers to further examine the interaction influence of servant leadership and individual difference on feedback-seeking behavior. Therefore, we aim to examine the moderating influence of traditionality on the relationship between servant leadership and outcomes. Farh et al. (1997) introduced the construct of Chinese traditionality to organizational science, it refers to “the extent that an individual endorses the hierarchy in relationships.” Indeed, leadership is about hierarchical relationships between the leader and the follower, followers with high scores on traditionalism are less likely than others to behave based on how leaders treat them (Spreitzer et al., 2005). However, that little is known about the role of contextual factors and individual differences influencing feedback-seeking at work. Therefore, in this study, we address this gap by examining traditionality as a moderator in the relationship between servant leadership and feedback-seeking behavior via *moqi*.

**FIGURE 1:**
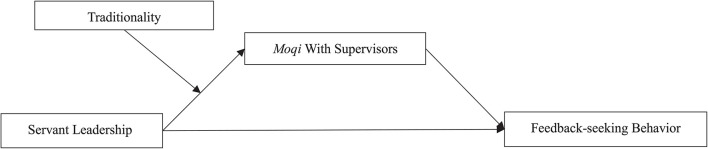
Hypothesized model.

In conclusion, our study aims to examine how contextual factors and individual differences influence employees’ feedback-seeking behavior, it has several theoretical and practical contributions in three aspects. First, this study further confirms whether and how employees’ feedback-seeking behavior was encouraged by servant leadership. Second, based on social information processing theory, we introduce *moqi* to our study, which fills the research gap on individuals’ feedback-seeking process and provides empirical support for the information interpreting mechanism of followers toward servant leaders. Third, traditionality was identified, so that we could combine the crucial antecedents of feedback-seeking behavior in our moderated mediation model, Figure 1 serves as a guide for specifying our model.

## Theoretical Background and Hypothesis Development

### Servant Leadership and Feedback-Seeking Behavior

Ashford and Cummings (1983) introduced the concept of feedback-seeking behavior (FSB) as “… [a] conscious devotion of effort toward determining the correctness and adequacy of behaviors for attaining valued end states.” Researchers have found leadership styles are significantly correlated with followers’ feedback-seeking behavior (e.g., Levy et al., 2002; Wang et al., 2016; Qian et al., 2017; Moss et al., 2020). Servant leadership is a desirable approach to leadership and have consistently demonstrated its ability to explain additional variance beyond other leadership styles in key outcomes, such as employee satisfaction, commitment, organizational citizenship behavior, in-role performance, and firm performance (Liden et al., 2008; Hoch et al., 2018; Sendjaya et al., 2019; Lee et al., 2020). Whether and how employees’ feedback-seeking behavior was encouraged by servant leadership remains unclear, we submit that social information processing theory can explain the relationship between servant leadership and feedback-seeking behavior.

Servant leadership is a multidimensional construct, involving forming relationships with followers, helping subordinates grow and succeed, empower followers, putting subordinates first, behaving ethically, and emotional healing, etc. (Ehrhart, 2004; Liden et al., 2008). It focuses on helping others and stresses bringing out the full potential of its followers primarily (Liden et al., 2015). Based on social information processing theory, followers use information from work contexts to understand leaders’ expectations and decide how to behave. They may consider interactions with a leader as a crucial aspect of the work environment and may behave based on their interpretation of leadership behavior. First, from the perspective of a leader, servant leaders are familiar with each follower’s actual abilities and needs through one-on-one communication, then they tend to assist followers in developing their potential pertinently. In addition, from the perspective of followers, if subordinates were treated with respect, compassion, trust, and patience at work, they are likely to feel psychologically safe (Chughtai, 2016) or self-efficacy, and then promote followers’ feedback-seeking behavior. As a result, they tend to seize the opportunity and seek feedback from leaders and perceive feedback from servant leaders as more credible and valuable. In this context, followers are more likely and frequently seek feedback about their work from the servant leader (Anseel et al., 2015; Huang et al., 2018). We predict the following:

*Hypothesis 1: Servant leadership is positively related to subordinates’ feedback-seeking behavior.*

### The Mediating Role of *Moqi* With Supervisors

Servant leadership involves forming good relationships with followers, scholars have suggested that servant leaders spend quality time and develop a high-quality exchange relationship with subordinates (Ehrhart, 2004). In this study, we focus on a key, but always overlooked, aspect of supervisor-subordinate relationships, i.e., subordinates’ *moqi* with supervisors (Zheng et al., 2019). Servant leadership contributes to followers’ perceived quality of the dyadic relationship between a leader and a member (van Dierendonck and Nuijten, 2011). As servant leaders focused on their followers’ needs, interests, and development, the relationships between leaders and subordinates are likely to be stably based on mutual trust and understanding (Kauppila et al., 2021). *Moqi* with supervisors as a key supervisor-subordinate relationship, from a subordinate-centric view, it could emphasizesubordinates’ tacit understanding of leaders’ work-related expectations and requirements (Zheng et al., 2019). Therefore, the emergence and development of *moqi* must base on mutual trust and understanding between leaders and subordinates, which could be facilitated by servant leadership.

Based on social information processing theory, followers use information get from leaders to understand leaders’ expectations, which in turn impact their bebavior in the work environment.Specifically, if subordinates were trusted, empowered, and treated with respect, compassion, and patience, they may obtain *moqi* with their supervisors and form a better understanding of their expectations and performance through the interaction with leaders. Thus, they are likely to feel psychologically safe and believe that they would not be punished or rejected because of seeking feedback (Yoshida et al., 2014; Chughtai, 2016). As a result, they tend to seize the opportunity and seek feedback from leaders without doubt and anxiety. In addition, servant leaders set a role model to subordinates, therefore, based on the *moqi* and mutual trust, followers may perceive feedback from servant leaders as more credible and valuable, and more proactively and frequently seek feedback from servant leaders. Therefore, servant leadership is likely to be associated with followers’ feedback-seeking behavior through *moqi* with supervisors. Accordingly, we hypothesized:

*Hypothesis 2: The relationship between servant leadership and subordinates’ feedback-seeking behavior was mediated by moqi with supervisors, servant leadership was positively related to moqi with supervisors and in turn positively related to subordinates’ feedback-seeking behavior.*

### The Moderating Role of Traditionality

Traditionality captures one’s endorsement of traditional hierarchical role relationships, which are prescribed by Confucian social ethics, such as emperor-subject, father-son, husband-wife, older brother-younger brother, and friend-friend (Farh et al., 2007). We propose that traditionality may moderate the mediated relationship between servant leadership and feedback-seeking behavior.

Leadership is about hierarchical relationships between the leader and the follower, for followers high in Chinese traditionality, leaders are treated like fathers and followers are treated like sons (Yang, 1993; Rarick, 2007). They assume the existence of a high level of power distance and collectivism, and are less likely to behave based on how leaders treat them (Spreitzer et al., 2005; Farh et al., 2007). This suggests that traditionalists are less sensitive and less likely to be suspicious of servant leader’s attitudes and behavior, because they tend to adjust their values more to conform to the traditional hierarchical values, and can’t put development ahead of their leader (Li et al., 2017). They firmly believe that meeting the expectations of the leader is a kind of responsibility and obligation, rather than an equal, mutual trust relationship. Therefore, high traditionality may negatively moderate the relationship between servant leadership and *moqi* with supervisors.

In contrast, employees with low traditionality tend to perceive the relationship with leaders as an equal relationship. It is not easy for them to accept unequal power distribution and differentiated status (Hui et al., 2004; Li et al., 2017). As a result, they are more proactive during the communication and interaction with supervisors in the work environment, and more sensitive to servant leader’s care and compassion. Thus, subordinates with low traditionality and servant leaders form a better mutual understanding of each expectation and interest through the interaction, it is easy for them to build mutual trust and understanding with servant leaders. Therefore, the positive influence of servant leadership on encouraging *moqi* with supervisors is more effective for those with low traditionality. Accordingly, we hypothesized:

*Hypothesis 3: Traditionality negatively moderates the relationship between servant leadership and moqi with supervisors, such that the relationship is stronger when traditionality is low.*

### The Moderated Mediation Model

As alluded above, it is logical to integrate H1-H3 into a moderated mediation model (Edwards and Lambert, 2007; Hayes, 2017). High traditionality can mitigate the positive influence of servant leadership on followers’ *moqi* with supervisors, and then influence employees’ feedback-seeking behavior.

For followers with high traditionality, the indirect relationship between servant leadership and feedback-seeking behavior via *moqi* with supervisors is weaker. Because they tend to conform to the traditional hierarchical obligation and authority, although their servant leaders put them first, and manifest respect and trust to them, they are dare not overstep these invisible boundaries. Therefore, it is not easy for them to build and develop *moqi* with supervisors. When they need to seek information feedback on performance, they are more likely to suppress their inner seeking motivation and behavior, that is, they tend not to seek feedback from leaders. In contrast, followers with low traditionality who develop high *moqi* with servant leaders would actively exchange ideas and suggestions to obtain more resources (Yao et al., 2019). Therefore, they are more likely to seek feedback from leaders more proactively. Accordingly, we hypothesized:

*Hypothesis 4: The indirect relationship between supervisors’ servant leadership and feedback-seeking behavior via moqi with supervisors is moderated by traditionality, such that the indirect relationship is weaker for those with high traditionality than for those with low traditionality.*

## Methods

### Participants and Procedures

The data for this study were collected from 10 companies in a range of different industries (e.g., energy industry, transportation industry, agricultural investment industry) operating in Henan province of the People’s Republic of China. We collected survey data through a two-wave questionnaire survey to minimize common method bias. The first round of data collection was in the December of 2020, and we asked the respondents to report their immediate supervisors’ servant leadership, traditionality, and demographic information of themselves. One month later, we invited them to report their *moqi* with supervisors and feedback-seeking behavior. All participants were assured of confidentiality and anonymity. Respondents’ gender, age and educational level, tenure, marital status, and job position were included as controls because of their potential relationships with variables. Among the 788 employees, 620 responded in the first wave, and 512 responded in the second wave, yielding a response rate of 64.9 percent. We used 440 responses due to invalid or incomplete responses. More specifically, it included 164 females (37.3%) and 276 males (62.7%), 50% of participants was 31–40 years old (*n* = 220), 77.3% of participants were married (*n* = 340). The tenure of half employees was 10 years above (*n* = 230, 52.3%). A total of 72.3% of the employees had a bachelor’s degree (*n* = 318). The majority were technical staff of various companies (*n* = 144, 32.7%), followed by department managers (*n* = 118, 26.8%), administrative staff (*n* = 70, 15.9%), chief executives (*n* = 22, 5.0%), and 86 (19.5%) reported other positions.

### Measures

**Servant leadership.** The 7-item measure of global servant leadership (SL-7) from Liden et al. (2015) was used to evaluate servant leadership. Bao et al. (2018) reported good reliability (higher than 0.80) for the Chinese version of this scale. Participants were asked to rate each item on a 5-point Likert scale from 1 (*strongly disagree*) to 5 (*strongly agree*). A sample item was “My leader makes my career development a priority.” In the present study, the internal consistency reliability of the SL-7 was 0.85.

**Traditionality.** To assess traditionality, we used the shortened version of the Chinese Individual Traditionality Scale (CITS; Yang et al., 1989) used by Farh et al. (1997). The scale includes five items (e.g., When people are in dispute, they should ask the most senior person to decide who is right), each item was rated on a 5-point Likert-type scale, ranging from 1 (*strongly disagree*) to 5 (*strongly agree*). In this study, the reliability of traditionality was 0.72.

**Subordinates’ *moqi* with supervisors.** Subordinates’ *moqi* with supervisors was measured using a subordinate-focused *moqi* scale developed by Zheng et al. (2019). The scale includes eight items (e.g., In day-to-day work situations, without explicit verbal communication or overt cues from my supervisor, I can understand his/her task requirements at work), each item was rated on a 5-point Likert-type scale, ranging from 1 (*strongly disagree*) to 5 (*strongly agree*). In this study, the Cronbach’s alpha was 0.94.

**Feedback-seeking behavior.** To measure feedback-seeking behavior, a five-item scale developed by VandeWalle et al. (2000) was employed. We utilized the Chinese version of this scale published by Zhang and Liao (2015). Participants were asked how frequently they sought feedback from their supervisors regarding their job performance and other four aspects and responded to the scale on a 5-point Likert scale from 1 (*never*) to 5 (*very frequently*). The Cronbach’s α for feedback-seeking behavior was 0.92.

## Results

### Confirmatory Factor Analysis

A series of CFAs was performed using the package ‘lavaan’ in R 4.0.2 (Rosseel, 2012; R Core Team, 2019) to examine the discriminant validity of the study variables. We compared a four-factor model with a three-factor model, a two-factor model, and a one-factor model. In the four-factor model, four key variables (servant leadership, *moqi* with supervisors, traditionality, feedback-seeking behavior) were treated as four independent factors. In the three-factor model, we loaded the items of servant leadership and *moqi* with supervisors on one factor. In the two-factor model, the items of servant leadership, *moqi* with supervisors, and traditionality were loaded on one factor. In the one-factor model, all variables were loaded on one factor. Models were assessed by the following fit indices: the χ^2^/*df*, CFI, TLI, RMSEA, and SRMR. As shown in Table 1, the hypothesized four-factor model fits the data well, χ^2^(269) = 726.14, CFI = 0.93, TLI = 0.92, RMSEA = 0.06, and SRMR = 0.05. Therefore, these results support the good discriminant validity of our measures.

**TABLE 1:** The result of confirmatory factor analyses.

Model	χ^2^	*df*	χ^2^*/df*	CFI	TLI	RMSEA [90% CI]	SRMR
Four-factor model^a^	726.14	269	2.70	0.93	0.92	0.06 [0.06, 0.07]	0.05
Three-factor model^b^	1670.30	272	6.14	0.78	0.76	0.11 [0.10, 0.11]	0.12
Two-factor model^c^	2052.86	274	7.49	0.72	0.70	0.12 [0.12, 0.13]	0.13
One-factor model^d^	3347.52	275	12.17	0.52	0.48	0.16 [0.15, 0.16]	0.15

*N* = 440.

*χ^2^, chi-square statistic; CFI, comparative fit index; TLI, Tucker-Lewis index; RMSEA, root mean square error of approximation; SRMR, standardized root mean square residual.*

*^*a*^Servant leadership, *Moqi*, Traditionality, FSB.*

*^*b*^Servant leadership + *Moqi*, Traditionality, FSB.*

*^*c*^Servant leadership + *Moqi*+ Traditionality, FSB.*

*^*d*^Servant leadership + *Moqi*+ Traditionality + FSB.*

### Descriptive Statistics

The descriptive statistics and Pearson correlations for the study variables are presented in Table 2. Employee’s perception about servant leadership is not significantly related to gender (*r* = 0.00, *p* > 0.05), age (*r* = 0.01, *p* > 0.05), and education (*r* = −0.08, *p* > 0.05). *Moqi* with supervisors is positively related to gender (*r* = −0.18, *p* < 0.001), and it is not significantly related to age (*r* = 0.10, *p* > 0.05) and education (*r* = −0.01, *p* > 0.05). Traditionality is positively related to his or her gender (*r* = −0.29, *p* < 0.001), age (*r* = 0.22, *p* < 0.001), and education (*r* = −0.15, *p* < 0.001). Employee’s feedback-seeking behavior is not presented significant relations with participants’ demographic variables.

**TABLE 2:** Means, standard deviations, and correlations among the variables.

	*M*	*SD*	1	2	3	4	5	6	7
1. Gender	1.38	0.49							
2. Age	2.02	0.81	−0.24***						
3. Education	1.95	0.52	0.03	−0.25***					
4. Servant leadership	4.28	0.64	0.00	0.01	–0.08				
5. *Moqi*	3.89	0.71	−0.18***	0.10	–0.01	0.25***			
6. Traditionality	3.57	0.69	−0.29***	0.22***	−0.15***	0.21***	0.21***		
7. FSB	3.82	0.87	–0.07	–0.01	0.01	0.24***	0.51***	0.09	

***p < 0.001.

Moreover, employee’s perception about servant leadership is positively related to their *moqi* with supervisors (*r* = 0.25, *p* < 0.001), traditionality (*r* = 0.21, *p* < 0.001), and feedback-seeking behavior (*r* = 0.24, *p* < 0.001). *Moqi* with supervisors is positive related to their traditionality (*r* = 0.21, *p* < 0.001) and feedback-seeking behavior (*r* = 0.51, *p* < 0.001). The correlations among traditionality and feedback-seeking behavior are not significant.

### Mediating Effect of *Moqi* With Supervisors

Using SPSS 26.0 Macro PROCESS (Model 4, Hayes, 2017), we analyzed the mediating role of *moqi* with supervisors between servant leadership and employee’s feedback-seeking behavior. Model 2 in Table 3 and Model 5 in Table 4 indicates that after controlling for the effect of employees’ gender, age, and education, servant leadership was found to be significantly and positively related to employee’s feedback-seeking behavior (*β* = 0.24, *p* < 0.001), and *moqi* with supervisors (*β* = 0.25, *p* < 0.001); When servant leadership and *moqi* with supervisors were simultaneously considered (Model 3 in Table 3), the relationship between servant leadership and employee’s feedback-seeking behavior was significant (*β* = 0.12, *p* < 0.01), which indicates that *moqi* mediated the relationship between servant leadership and employee’s feedback-seeking behavior. Hypothesis 1 was supported. To further clarify the mediation effect, we used a bootstrap procedure with 5,000 samples to produce a confidence interval (CI) for the indirect effect. The indirect effect of servant leadership (via *moqi* with supervisors) on feedback-seeking behavior (indirect effect = 0.12, SE = 0.02, 95% CI = [0.08, 0.17]) was statistically significant. Overall, the indirect effect of servant leadership on employee’s feedback-seeking behavior via *moqi* with supervisors was significant, these results provide support for Hypothesis 1 and Hypothesis 2.

**TABLE 3:** Hierarchical regression: the mediating effect of servant leadership on FSB.

Variables and statistic	FSB		FSB			
	Model 1		Model 2		Model 3	
	β	*se*	β	*se*	β	*se*
Gender	–0.07	0.09	–0.07	0.09	0.00	0.08
Age	–0.02	0.05	–0.02	0.05	–0.05	0.05
Education	0.00	0.08	0.02	0.08	0.01	0.07
Servant leadership			0.24***	0.06	0.12**	0.06
*Moqi*					0.48***	0.05
F	0.74		7.27***		32.38***	
R^2^	0.01		0.06		0.27	

**p < 0.01; ***p < 0.001.

**TABLE 4:** Hierarchical regression: the moderation effects of Traditionality.

Variables and statistic	*Moqi*		*Moqi*			
	Model 4		Model 5		Model 6	
	β	*se*	β	*se*	β	*se*
Gender	−0.16**	0.07	−0.16***	0.07	−0.13**	0.07
Age	0.06	0.04	0.06	0.04	0.06	0.04
Education	0.01	0.07	0.03	0.06	0.04	0.06
Servant leadership			0.25***	0.05	0.22***	0.05
Traditionality					0.11*	0.05
Servant leadership* Traditionality					−0.11*	0.07
F	5.04**		11.76***		9.95***	
R^2^	0.03		0.10		0.12	

*p < 0.05; **p < 0.01; ***p < 0.001.

### Moderated Mediation Effects

Using Hayes (2017) PROCESS macro (Model 7), we assessed our whole moderated mediation model. Hypothesis 3 states that traditionality moderates the relationship between servant leadership and *moqi* with supervisors, and Hypothesis 4 states that traditionality moderates the indirect relationship between servant leadership and feedback-seeking behavior via *moqi* with supervisors.

As Model 6 shown in Table 4, the interaction term of servant leadership and traditionality was statistically significant (*β* = −0.11, *p* < 0.05). Traditionality moderated the mediation effect of *moqi* with supervisors on servant leadership and employee’s feedback-seeking behavior. Thus, H3 was supported. Then, to examine the whole research model, we conducted a bootstrapping procedure with 5,000 samples to test the conditional indirect effect of servant leadership through *moqi* with supervisors on employee’s feedback-seeking behavior at both high and low levels of traditionality. As shown in Table 5, the conditional indirect effect of servant leadership on feedback-seeking behavior via *moqi* with supervisors was statistically significant when traditionality was low (−1 SD, indirect effect = 0.16, SE = 0.03, 95% CI = [0.10, 0.22]) compared with when traditionality was high (+1 SD, indirect effect = 0.06, SE = 0.04, 95% CI = [−0.02, 0.14]). Hypothesis 4 thus received support.

**TABLE 5:** Results for conditional indirect effect analysis.

Outcome variable	Level of traditionality	Indirect effect	SE	LLCI	ULCI
FSB	−1SD	0.16	0.03	0.10	0.22
	Mean	0.11	0.03	0.06	0.16
	+1SD	0.06	0.04	–0.02	0.14

*Bootstrap size = 5,000.*

**FIGURE 2:**
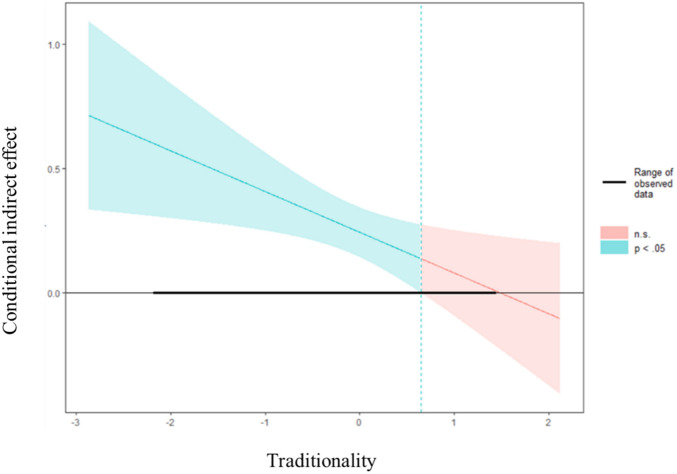
Johnson–Neyman regions of significance for the conditional effect of servant leadership at values of traditionality.

Finally, we used the Johnson–Neyman technique to identify the regions in the range of the moderator variable where the effect of servant leadership on an employee’s feedback-seeking behavior is statistically significant (Johnson, 2008; Hayes and Matthes, 2009). The results may help provide more preventive interventions and pointed suggestions. Figure 2 indicates that when the traditionality is smaller than 1.026 (on a 5-point scale), that is, the blue area on Figure 2, the indirect effect is significantly different from zero, which is consistent with the previous hypotheses, indicating the effect of servant leadership on employee’s feedback-seeking behavior via *moqi* is significant and negatively moderated by traditionality.

## Discussion

This research investigates the link between servant leadership and feedback-seeking behavior to extend our understanding of the effect of servant leadership, as well as the internal mechanism behind it. The purpose of this study was to investigate whether the servant leadership and subordinates’ feedback-seeking behavior was mediated by *moqi* with supervisors and whether these direct and indirect associations were moderated by traditionality. We found that servant leadership was positively related to *moqi* with supervisors and in turn positively related to feedback-seeking behavior. Low traditionality enhanced the effect of servant leadership on *moqi* with supervisors and the indirect effect of servant leadership on feedback-seeking behavior via *moqi* with supervisors.

First, servant leadership has a significant positive impact on employees’ feedback-seeking behavior, which supports H1. Previous researches have demonstrated leaders’ impact on employees’ feedback-seeking, Levy et al. (2002) invited 132 students who were presented with a vignette describing either a transformational or a transactional leader, and found transformational leader was significantly related to higher feedback-seeking intentions. Wang et al. (2016) used data from a survey of 205 supervisor-subordinate dyads in a high-technology communications company in China, they also indicated that transformational leadership was positively related to employees’ feedback seeking. Drawing on social learning theory, Moss et al. (2020) used a sample of 249 followers across two waves of data collection and their findings indicated ethical leadership has a positive relationship with followers’ feedback-seeking behavior. In addition, using data from 64 supervisors and 265 of their immediate employees in China, Qian et al. (2017) found a positive relationship between ethical leadership and feedback-seeking from both ethical leaders and coworkers. Compared with transformational and ethical leadership, servant leadership could better explain a wide range of outcomes (Hoch et al., 2018; Lee et al., 2020). However, there are seldom studies examined the influence of servant on feedback-seeking behavior, most studies focused on engagement (De Clercq et al., 2014), organizational citizenship behavior (OCB) (Liden et al., 2014), task performance (Chen et al., 2015), and creativity (Yoshida et al., 2014). To investigate whether and how employees’ feedback-seeking behavior was encouraged by servant leadership, with the insights provided by the SIPT, the findings of our study verify the impact of servant leadership on employees’ feedback-seeking behavior.

Second, we test the mediating effect between servant leadership and feedback-seeking behavior. The results indicated that subordinates’ *moqi* with supervisors mediated the positive effects of servant leadership on feedback-seeking behavior, which supports H2. Previous researches have examined the mediating mechanism of other constructs, for example, Wang et al. (2016) indicated that trust in leader mediated the relationship between transformational leadership and employees’ feedback seeking. Moss et al. (2020) conducted a survey from both moral and non-moral ways and found that duty orientation would mediate the relationship between ethical leadership and feedback-seeking/feedback-avoiding behavior. From the perspective of supervisor-subordinate relationships, leader-member exchange (LMX) mediated the positive relationship between ethical leadership and feedback-seeking from both ethical leaders and coworkers. At the same time, we focus on a key, but always overlooked kind of supervisor-subordinate relationships, i.e., subordinates’ *moqi* with supervisors. Similarly, The outcomes of our research are also supported by the earlier studies (Chughtai, 2016), which indicated that *moqi* helps subordinates form a better understanding of their expectations and performance through the interaction with leaders, so that they could have confidence and psychological safety to seek feedback without hesitation.

Third, this study verifies that the negative moderating effect of traditionality, which supports H3 and H4, and constitutes an original contribution in the context of an emerging or developing country like China. Previous studies found that traditionality positively moderates the relationship between leadership and outcomes, Liu et al. (2013) based on two-phase data from a sample of 312 supervisor-subordinate dyads in China, indicated that subordinates’ traditionality strengthened the relationships between ethical leadership and task performance and organizational citizenship behavior. Li et al. (2014) proposed that Chinese traditionality strengthens the positive effects of authentic leadership on in-role performance, creativity, and organizational citizenship behavior. However, our study found that traditionality mitigated the positive impact of servant leadership on feedback-seeking behavior. The reason might be that followers with high scores on traditionalism are less likely than others to behave based on how leaders treat them (Spreitzer et al., 2005), servant leadership trust and respect the employees, they could form high-quality interpersonal relationships with their followers, while high traditionalists tend to respond more favorably to respect superior-subordinate hierarchical relationships than low traditionalists. Therefore, high levels of traditionality negatively moderate the relationship between servant leadership and employees’ feedback-seeking behaviors. For followers with high traditionality, they tend to conform to the traditional hierarchical obligation and authority, they are dare not overstep these invisible boundaries. It is not easy for them to build and develop *moqi* with supervisors and seek feedback from leaders. In contrast, followers with low traditionality who develop high *moqi* with servant leaders would actively exchange ideas and suggestions to obtain more resources (Yao et al., 2019).

### Theoretical Contributions

As mentioned above, this study explores the mechanism and boundary conditions of servant leadership on employees’ feedback-seeking behavior. The main theoretical contributions are as follows: First, our results trying to investigate the antecedents of feedback-seeking literature from the perspective of servant leadership style. Previous studies have evidenced that leadership style significantly influence employee feedback-seeking behavior, involving transformational leadership (Levy et al., 2002; Wang et al., 2016) and ethical leadership (Qian et al., 2017; Moss et al., 2020), little is known about the impact of servant leadership. In addition, researchers have evidenced the incremental validity of servant leadership over other leadership styles, it could better explain a wide range of outcomes. Although Chughtai (2016) found that servant leadership can promote employees’ negative feedback-seeking behavior, feedback-seeking behavior includes positive feedback and negative feedback, the relationship between servant leadership and overall feedback-seeking behavior remains unclear, we addressed this shortcoming by clarifying and confirming employees’ feedback-seeking behavior was encouraged by servant leadership.

Second, based on the social information processing theory, this paper further reveals that the mechanism of servant leadership on followers’ feedback-seeking behavior. Studies have found that *moqi* with supervisors has not been fully explored as a mediator between leadership and outcomes (Zheng et al., 2019), we are the first to employ social information processing theory to explain the transmission mechanism of *moqi* with supervisors in the relationship between servant leadership and feedback-seeking behavior. Our study confirmed the servant leadership can indirectly affect followers’ feedback-seeking behavior through their *moqi* with supervisors. That is, *moqi* with supervisors is a new type of mediator that links servant leadership with employee feedback-seeking behavior, which opens the black box of the relationship between servant leadership and employees’ feedback-seeking behavior.

Third, this study clarifies the boundary conditions of the relationship between servant leadership and employees’ feedback-seeking behavior. Consistent with previous studies (Ashford et al., 2003; Anseel et al., 2015), we found that contextual factors and individual differences are crucial antecedents of employees’ feedback-seeking behavior. This research has constructed a moderated mediation model by the addition of traditionality as a moderator to explore its moderating effect on the relationship between servant leadership, *moqi* with supervisors, and feedback-seeking behavior. It is also worth noting that our model is largely derived from Chinese culture since *moqi* with supervisors and traditionality are most frequently observed in Chinese societies. Our results found that traditionality plays a negative moderating role in the relationship between servant leadership and *moqi* with supervisors, and negatively moderated the mediating effect of *moqi* with supervisors in the relationship between servant leadership and feedback-seeking behavior. Therefore, this study combines servant leadership theory with Chinese traditional values to explore boundary conditions of the relationship between servant leadership and employees’ feedback-seeking behavior, which deepens our understanding of the relationship between them.

### Practical Implications

The findings of this research can have important implications for organizations. Results indicated that it is worthwhile to implement servant leadership in managerial practice since servant leadership can inspire followers to seek feedback proactively, collect information to improve their performance, subsequently team and organizational performance. Therefore, first, organizations should select managers with servant leadership skills, including develop the serving and empowering skills of leaders through providing training and development programs, so that supervisors can learn how to encourage employees’ feedback-seeking behavior, especially as it relates to their interaction relationship with supervisors. In addition, organizations also could create an altruistic atmosphere, so that leaders can adopt servant leadership philosophy, emphasize caring and concerning followers, and put development and improvement of employees first, which facilitates employee feedback-seeking behavior through *moqi* with them.

Second, our study provides valuable insight into how servant leaders can facilitate employees’ feedback-seeking behavior. It is suggested that supervisors can play an important role in facilitating employees’ feedback-seeking behavior by developing the *moqi* with them. Therefore, organizations may help individuals recognizing the importance of *moqi*, which will hopefully motivate them to be more sensitive to the feedback offered by supervisors. As for supervisors, they should be aware of their roles in cultivating feedback-seeking, and identify the needs and interests of subordinates, strengthen communication with their subordinates proactively, and participate in team-building activities with subordinates, then cultivate subordinates’ *moqi* with them through mutual understanding and support. When recruiting and selecting leaders, relevant tests can be carried out to test the matching degree between supervisors and subordinates, which helps to cultivate *moqi* between supervisors and subordinates in the future.

Third, contextual factors and individual differences are crucial antecedents of employees’ feedback-seeking behavior, so we can not ignore the importance of the traditionality of Chinese employees. Therefore, leaders should adopt differentiated management for employees with different traditionality levels. For employees with low traditionality, leaders need to strengthen their interpersonal interaction with subordinates, establish high-quality leader-member exchange and thereby improve subordinates’ *moqi* with supervisors. For organizations that employ quite a lot of employees who have high traditionality, or organizations that have a traditionalist culture like China, leaders should consider other strategies for encouraging feedback-seeking behavior. For example, they should consider other strategies for encouraging feedback-seeking behavior. One alternative strategy is to cultivate subordinates’ *moqi* with supervisors, emphasize employees’ duty to work roles and loyally to their supervisors, cultivate mutual trust and understanding between them simultaneously, which is not only consistent with traditional Chinese culture but also shorten the distance between the leaders and subordinates.

### Limitations and Future Directions

As with any research, our research has several limitations and, it does provide several insights for future research. First, although our research model is built on solid theories, our cross-sectional research design may generate a concern of causality in results. In the future, longitudinal designs should be conducted to provide evidence of reliable causalities. For example, a previous study found subordinates’ feedback-seeking positively predicted their subsequent perceptions of *moqi* with a supervisor (Zheng et al., 2019), it is essential to adapt longitudinal designs to investigate reliable causalities.

Second, although the research was conducted in Chinese culture and, the generalizability of our findings was limited. We only examined a relatively small sample of companies, it is worthwhile to extend our findings using other types of organizations to generate a more generalization result. For instance, civil servants in the public sector may be more conform to traditional responsibilities and hierarchical values.

Finally, future research is needed to test our findings in other contexts as well. Obviously, contrary to western countries, Confucianism has persisted as a major cultural force for thousands of years in China, traditional hierarchical role relationships play an important role in the workplace. Given the contextual factors and individual differences are crucial antecedents of employees’ feedback-seeking behavior, we suggest that this study be tested and compared in other contexts.

## Conclusion

Our study of feedback-seeking behavior integrates SIPT with a leader-follower *moqi* perspective to provide a comprehensive analysis of how servant leadership contributes to feedback-seeking behavior. The present study indicates that leaders can put subordinates first and focus on employees’ development, which has a significant influence on employee feedback-seeking behavior through the *moqi* followers developed with them. Moreover, this is the first and novel research indicating that contextual factors and followers’ *moqi* with leaders could enhance feedback-seeking behavior and that traditionality trait reduces this positive effect. Specifically, our research suggests that high traditionality buffer the positive effect of servant leadership first on *moqi* and then on feedback-seeking behavior.

The above-mentioned outcomes have useful implications for academic researchers and managers. Organizations and managers may help subordinates recognizing the importance of *moqi and* cultivate *moqi* with them through mutual understanding, in turn, motivate them to be more sensitive to the feedback. Just because the level of this study is amateur, it is the first study to combine contextual factors and indifference to examine the relationship between servant leadership and feedback-seeking behavior. In the future, such a study must be tested at the global level, and it must be applied in both eastern countries and western countries.

## Data Availability Statement

The original contributions presented in the study are included in the article/supplementary material, further inquiries can be directed to the corresponding author/s.

## Ethics Statement

Ethical review and approval was not required for the study on human participants in accordance with the local legislation and institutional requirements. Written informed consent was not provided because the authors declare that they strictly adhered to the APA guidelines on ethical research practices. Written informed consent for participation was not required for this study in accordance with the national legislation and the institutional requirements.

## Author Contributions

All authors listed have made a substantial, direct and intellectual contribution to the work, and approved it for publication.

## Conflict of Interest

The authors declare that the research was conducted in the absence of any commercial or financial relationships that could be construed as a potential conflict of interest.

## Publisher’s Note

All claims expressed in this article are solely those of the authors and do not necessarily represent those of their affiliated organizations, or those of the publisher, the editors and the reviewers. Any product that may be evaluated in this article, or claim that may be made by its manufacturer, is not guaranteed or endorsed by the publisher.
